# A Rapid and Cost-Effective Identification of Invertebrate Pests at the Borders Using MinION Sequencing of DNA Barcodes

**DOI:** 10.3390/genes12081138

**Published:** 2021-07-27

**Authors:** Shamila Weerakoon Abeynayake, Sonia Fiorito, Adrian Dinsdale, Mark Whattam, Bill Crowe, Kate Sparks, Paul Richard Campbell, Cherie Gambley

**Affiliations:** 1 Plant Innovation Centre, Plant Import Operations, Biosecurity Plant Division, Department of Agriculture, Water and the Environment (DAWE), Canberra, ACT 2601, Australia; Sonia.Fiorito@agriculture.gov.au (S.F.); Adrian.Dinsdale@agriculture.gov.au (A.D.); Mark.Whattam@awe.gov.au (M.W.); 2 Department of Animal, Plant and Soil Sciences, Centre for AgriBiosciences, La Trobe University, Bundoora, VIC 3086, Australia; 3 Operational Science and Surveillance, Science and Surveillance Group, Biosecurity Plant Division, Department of Agriculture, Water and the Environment (DAWE), Canberra, ACT 2601, Australia; Bill.Crowe@awe.gov.au (B.C.); Kate.Sparks@awe.gov.au (K.S.); 4 Microbiology and Entomology, Biosciences, Department of Agriculture and Fisheries, Queensland Government (DAF, QLD), Brisbane, QLD 4001, Australia; Paul.campbell@daf.qld.gov.au

**Keywords:** cytochrome c oxidase subunit I, MinION sequencing, invertebrate pests, biosecurity

## Abstract

The rapid and accurate identification of invertebrate pests detected at the border is a challenging task. Current diagnostic methods used at the borders are mainly based on time consuming visual and microscopic examinations. Here, we demonstrate a rapid in-house workflow for DNA extraction, PCR amplification of the barcode region of the mitochondrial cytochrome oxidase subunit I (*COI*) gene and Oxford Nanopore Technologies (ONT) MinION sequencing of amplified products multiplexed after barcoding on ONT Flongle flow cells. A side-by-side comparison was conducted of DNA barcode sequencing-based identification and morphological identification of both large (>0.5 mm in length) and small (<0.5 mm in length) invertebrate specimens intercepted at the Australian border. DNA barcode sequencing results supported the morphological identification in most cases and enabled immature stages of invertebrates and their eggs to be identified more confidently. Results also showed that sequencing the *COI* barcode region using the ONT rapid sequencing principle is a cost-effective and field-adaptable approach for the rapid and accurate identification of invertebrate pests. Overall, the results suggest that MinION sequencing of DNA barcodes offers a complementary tool to the existing morphological diagnostic approaches and provides rapid, accurate, reliable and defendable evidence for identifying invertebrate pests at the border.

## 1. Introduction

The global spread of invasive pests is expected to increase over the coming decades, requiring more effective surveillance tools for biosecurity compliance. Invertebrates are a large and diverse group [1], including joint-legged arthropods, such as mites, aphids, spiders and fleas. Invertebrate pests can vector severe disease agents and cause significant yield losses in agronomically important crops. In Australia, invertebrate pests are estimated to cause in excess of $300 million in yield loss per annum [2]. 

Many invertebrate pests belong to taxonomic groups that are not well studied. For example, less than 20% of species in the class Arachnida are known and described [3]. Most mites are small individuals that are difficult to observe without specialist expertise to prepare and identify under high magnification. For instance, adult mites range in size from 0.5 mm to 2 mm in length, with their nymphal, larval and egg stages significantly smaller (0.02–0.03 mm in diameter) [4]. Therefore, the identification of such species and their immature stages based on microscopic observation is extremely difficult, time consuming and requires significant expertise. Recent advances in DNA sequencing technologies allow the use of genetic markers (DNA barcodes) to support and confirm morphological evidence for the identification of invertebrate pests and their immature life stages. DNA barcoding is widely used in the identification and taxonomic analysis of species.

The variable region of the mitochondrial *cytochrome oxidase subunit I* (*COI*) gene is recognised as a universal barcode for insect species identification, and millions of *COI* barcode sequences are publicly stored in the National Centre for Biotechnology Information (NCBI, https://www.ncbi.nlm.nih.gov/, accessed on 1 August 2020) and Barcode of Life Data System (BOLD, http://www.boldsystems.org/, accessed on 15 September 2020) databases [5]. Most DNA barcodes are currently generated by Sanger and second-generation sequencing technologies, such as Illumina, MiSeq and HiSeq, which require access to well-equipped molecular biology laboratories and specialised equipment. The third-generation sequencing platforms, such as the Oxford Nanopore Technologies (ONT, Oxford, UK) and Pacific Bio-sciences (PacBio, Menlo Park, CA, USA) “Sequel”, have a long-read sequencing capacity to generate full-length DNA barcodes. MinION is the smallest and most user-friendly sequencer currently available, and can run via a USB connected to a standard computer. The lower initial cost and portability of MinION may permit diagnostics to be conducted at the borders [6]. Recent studies have demonstrated the use of MinION-based sequencing approaches for species identification [7,8]. These approaches include DNA barcoding, whole genome sequencing, metagenome sequencing, transcriptome sequencing, metatranscriptome sequencing and mitochondrial genome sequencing [7,8,9,10,11]. However, DNA barcoding is the fastest approach for species identification, as genomic and transcriptomic sequencing approaches require longer data analysis workflows that require time to assemble sequences before the identification is possible. MinION sequencing offers a rapid and cost-effective approach to analysing smaller samples, making it more suitable for day-to-day border detections. Unlike Illumina and other second-generation sequencing technologies, MinION has the capability to generate full-length DNA barcodes [11]. Recent studies have identified MinION as a promising diagnostic tool for the identification of invertebrate pests [11,12,13]. Using MinION as a diagnostic tool requires the successful implementation of a workflow with three main factors [13]: DNA extraction from the specimen, PCR amplification of DNA barcodes and the generation of consensus sequences.

Extraction methods to obtain sufficient DNA for PCR from an individual invertebrate and other life stages is an important initial step for DNA barcode sequencing. This is a challenging task, especially when the specimen size is very small. Previous studies have used numerous extraction methods, such as EZNA Insect DNA kit (Omega Bio-Tek, Norcross, GA, USA), CHELEX (Bio-Rad Laboratories, Gladesville, NSW, Australia) and QuickExtract^TM^ DNA Extraction Solution (Lucigen Cooperation, Middleton, WI, USA) [14,15], from small invertebrate species. The non-destructive DNA extraction methods allow the specimens to be preserved post-DNA extraction [16,17,18]. However, these different extraction methods have advantages and disadvantages. 

The aim of the current study was to develop a rapid in-house workflow for DNA extraction, PCR amplification of the *COI* barcode and MinION sequencing of amplified products multiplexed on ONT Flongle flow cells. Firstly, DNA extraction protocols were optimised to extract sufficient amounts of DNA for PCR from an individual specimen. A DNA extraction protocol that left invertebrate exoskeletons intact (non-destructive) was optimised in addition to a destructive protocol where a specimen was homogenised in extraction buffer. PCR-amplified barcodes from 12 to 24 specimens were multiplexed together on an ONT Flongle flow cell for sequencing in order to reduce the associated cost per specimen. The suitability of MinION as a diagnostic tool was further assessed using side-by-side comparative identifications of invertebrate specimens intercepted at the border via DNA barcode sequencing and morphological examination. The suitability of ONT rapid sequencing as a quick and cost-effective approach for identifying invertebrate pests was assessed by sequencing PCR-amplified *COI* barcodes.

## 2. Materials and Methods

### 2.1. Specimen Collection

The invertebrate pest specimens analysed were from either border interceptions by the Science and Surveillance Group (SSG) at the Department of Agriculture, Water and the Environment (DAWE) or agricultural field samples collected through crop survey activities of the Department of Agriculture and Fisheries, Queensland Government (DAF, QLD). All specimens were stored in 80% ethanol at 4 °C until DNA was extracted.

### 2.2. DNA Extraction

QuickExtract^TM^ DNA Extraction Solution (Lucigen Cooperation, Middleton, WI, USA) was used as a non-destructive method for extracting DNA from an individual invertebrate pest (>0.5 mm in length) and used in a PCR reaction. An individual invertebrate pest was transferred to a 0.2 mL PCR tube, residual ethanol was removed and 10 µL of QuickExtract^TM^ DNA Extraction Solution was added and then incubated at 65 °C for 20 min and 98 °C for 2 min in a thermal cycler (T100, Bio-Rad Laboratories, Gladesville, NSW, Australia). After incubation, the extraction solution, now containing the DNA, was transferred to a sterile 1.5 mL Eppendorf tube and stored at −20 °C. To extract enough DNA for PCR from different life stages of small invertebrates (<0.5 mm in length; adult, nymph or egg), a destructive extraction method was needed. For this, an individual specimen was transferred to a sterile petri dish and air-dried. QuickExtract^TM^ DNA Extraction Solution (3 µL) was then added directly on to the specimen and homogenised using a 0.6 × 32 mm sterile syringe tip (AGANI^TM^ needle, Terumo, Hamburg, Germany). The homogenised solution was then transferred to a 0.2 mL PCR tube and incubated as per the non-destructive method above.

### 2.3. Amplification of DNA Barcode

PCR was performed using LCO1490 and HCO2198 universal *COI* primers (Table 1) [19] to amplify a DNA fragment of 709 bp from the *COI* gene. Each 50 µL PCR reaction consisted of 1 µL of DNA extract, 10 µL of 5X Phusion HF buffer (ThermoFisher Scientifics, Waltham, MA, USA), 1 µL of 10 mM dNTPs (ThermoFisher Scientifics), 0.5 µM of each primer (Integrated DNA Technologies, IDT, Singapore) and 0.5 µL of Phusion Hot Start II DNA Polymerase (2 U/µL, ThermoFisher Scientifics). After initial denaturation of DNA at 98 °C for 30 s, reactions were incubated through 35 cycles of 10 s at 98 °C, 30 s at 40 °C, and 30 s at 72 °C, followed by a final extension step at 72 °C for 10 min. Reactions were analysed by gel electrophoresis by loading 5 µL of PCR product on a 1% agarose E-Gel (ThermoFisher Scientifics). When templates provided insufficient quantities of product from a single amplification reaction, 1 µL from the first reaction was used as the template for a second amplification reaction. 

### 2.4. Sequencing COI Amplicons

Sequencing *COI* amplicons were carried out using MinION sequencing technology. Sanger sequencing was also carried out on 20 amplicons for comparison of Sanger and MinION consensus reads of the same sample. PCR-amplified products (*COI* barcodes) were purified using the Wizard PCR Clean-Up System (Promega, Madison, WI, USA) according to the manufacturer’s instructions. When non-specific bands were also amplified, the expected band was purified using E-Gel CloneWell II 0.8% agarose gel (ThermoFisher Scientifics) according to the manufacturer’s instructions. The purified PCR products were quantified using Qubit 2.0 fluorimeter and the Quanti-iT™ dsDNA assay kit (ThermoFisher Scientifics) according to the manufacturer’s instructions. The purified PCR products (30 ng from each amplified product) were sent to the Australian Genome Research Facility (AGRF, Melbourne, Australia) for Sanger sequencing. MinION sequencing libraries were prepared using SQK-LSK109 DNA sequencing kit (ONT, Oxford, UK) according to the manufacturer’s instructions. Preparation of DNA ends for adapter attachment was carried out using 200 fmol of purified PCR product. The libraries were barcoded using Native Barcoding Expansions kits 1–12 (ONT, EXP-NBD 104) and 13–24 (ONT, EXP-NBD 114) before the ligation of sequencing adapters and multiplexing on a Flongle flow cell for MinION sequencing. Equimolar amounts of each barcoded sample were pooled into a 1.5 mL Eppendorf DNA LoBind tube, ensuring that the pooled sample contained 200 fmol of library in total. The pooled barcoded sample was used for sequencing adapter ligation according to the manufacturer’s instructions. The various clean-up procedures at the end-preparation and ligation stages used AMPure beads (Beckmann Coulter, Brea, CA, USA) according to the manufacturer’s instructions. Flongle flow cells were primed with a mix of Flush tether (ONT, FLT) (3 µL) and Flush buffer (ONT, FB) (117 µL), and 3–20 fmol of the total sequencing library was loaded through the sample port according to the manufacturer’s instructions. The sequencing was carried out using MinION sequencer. To sequence amplicons using the rapid sequencing kit (ONT, SQK-RAD004), library preparation was performed in a volume of 10 µL per reaction. Each reaction consisted of 1 µL of PCR-amplified product, 6.5 µL of nuclease-free water and 2.5 µL of fragmentation mix (ONT, FRA). After incubation of the mix at 30 °C for 1 min followed by 80 °C for 1 min, 1 µL of rapid adapter (ONT, RAP) was added to the tube and incubated for 5 min at room temperature. The sequencing library (1 µL) was then loaded onto a Flongle flow cell following the manufacturer’s instructions (ONT) and a 1 h run was conducted using the standard settings of the MinION sequencer.

### 2.5. Bioinformatic Analysis 

After MinION sequencing, raw reads were basecalled and demultiplexed using MinIT, pre-configured with required MinKNOW, Guppy and EPI2ME software (ONT). After basecalling, the data files were analysed using Geneious Prime software (Biomatters, Auckland, NZ) and sorted by length, and reads were selected for the expected size (approximately 709 bp). Three consensus sequences were generated from each specimen after multiple sequence alignments of 40 individual reads per each consensus sequence using the Geneious Prime bioinformatics software platform. The final consensus sequence was generated after aligning all three of the consensus sequences. In order to generate the consensus sequences using the reads from rapid sequencing, first the contigs were prepared using Geneious Prime, and then the longest and the best quality contigs were selected as consensus sequences. The consensus sequences were used to blast against the nucleotide sequences in NCBI and BOLD databases for identification of the closest matching reference recodes.

## 3. Results and Discussion

### 3.1. Identification of Invertebrates 

**Figure 1:**
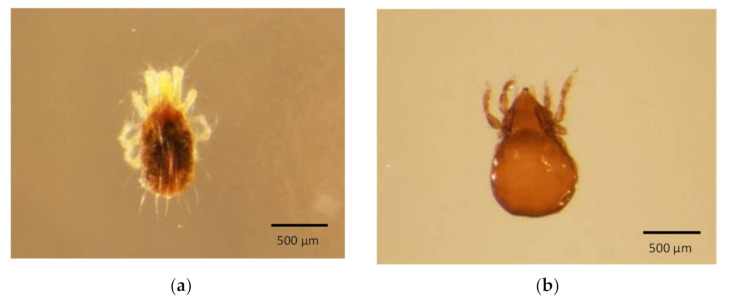
Two mite specimens post DNA extraction using non-destructive QuickExtractTM DNA extraction-based method: (**a**) *Tetranychus ludeni*; (**b**) *Reductobates bullager*.

The non-destructive DNA extraction method using QuickExtract^TM^ DNA Extraction Solution successfully amplified *COI* fragments from large insects (>0.5 mm in length), such as thrips and khapra beetle, and small invertebrates (<0.5 mm in length), such as mites. This non-destructive DNA extraction method allowed the specimens to be preserved post-DNA extraction. For example, exoskeletons of two adult mites without a disruption of morphological features after DNA extraction are shown in Figure 1. DNA extraction using this enzyme-based method required a heat treatment (65 °C for 20 min) in order for the lysis of epithelial cells and cellular structures to release DNA [20] followed by an incubation at 98 °C for 2 min to inactivate enzymes. However, the quality and the quantity of DNA obtained from this method can vary between specimens. Non-destructive methods did not provide sufficient DNA for PCR from some of the smaller specimens. Insufficient amounts of DNA extracted from these specimens led to amplifying untargeted template DNA. Therefore, in this study, a destructive method was also optimised by homogenising the specimen in an extraction solution in order to expose more epithelial cells and cellular structures to increase the DNA quantity. This destructive method was used to amplify *COI* fragments from most of the small specimens, such as nymphs and eggs. 

MinION barcode sequencing of PCR-amplified DNA could identify specimens of large insects (>0.5 mm in length), such as the khapra beetle (*Trogoderma granarium*), carpet beetle *(Trogoderma anthrenoides),* warehouse beetle (*Trogoderma variabile)* and two species of thrips (*Franklinella occidentalis* and *Franklinella shultzei*) to species level with an over 97% sequence identity (Table 2). NCBI accession numbers of the closest matching reference records are shown in Supplementary Table S1.

The khapra beetle is one of the world’s most destructive stored grain pests, and a major quarantine threat to global biosecurity [21]. The visual and microscopic identification of khapra beetle larvae and adults is a difficult task due to the morphological similarity with other *Trogoderma* species. *Trogoderma* *anthrenoides* and *Trogoderma* *variabile* are not considered as seriously as *Trogoderma* *granarium*, but they are also major pests that occasionally cause damage to grains and other food substances [22]. 

The early determination of invertebrates intercepted on avocadoes by microscopic observation could identify only 10 out of 100 individual specimens to genus or lower taxonomic levels. Most of the specimens were identified to order/family or higher taxonomic levels by microscopic observation. Analysis of the same specimens by *COI* barcode sequencing using MinION identified 24% of the specimens to genus or lower taxonomic levels, and in 34% of the specimens, MinION sequencing of *COI* barcode would have enhanced or supported biosecurity decision making (Supplementary Table S2). Some of the examples are shown in Table 3.

The Arachnida specimens identified to species level with over 97% sequence identity included *Tyrophagus curvipenis*, *Tetranychus urticae*, *Tetranychus ludeni* and *Aculops lycopersici*. *Tyrophagus curvipenis* can cause damage to stored food and economic plants [23,24]. *Aculops lycopersici* is a widespread pest of solanaceous plants, such as tomato, eggplant and capsicum [25,26]. *Tetranychus urticae* is considered to be the most important tetranychid pest species, causing yield losses in important crops, including vegetables, fruits and ornamentals [27,28]. Other members of the genus *Tetranychus* can also cause significant damage to a variety of crop species [29,30]. The predatory mite *Phytoseiulus persimilis*, known as the biological control agent of spider mites [31], was also identified to species level. 

Most of the mite specimens showed 80–95% identity to their closest matching reference record. In most cases, the closest matching reference record supported the morphological identification (Supplementary Table S2). Both morphological and DNA barcode sequencing-based identifications showed that most mite specimens belong to the order *Sarcoptiformes*. Over 85% of the egg specimens were identified to lower taxonomic levels by DNA barcode MinION sequencing compared to morphological identification. For example, the egg specimens that were numbered 8 and 10 (Supplementary Table S2) were identified to class and order levels, respectively, by morphological analysis, and later identified to species and genus levels, respectively, by DNA barcode sequencing using MinION technology. The results, however, showed some discrepancies between morphological and molecular identifications. One egg specimen (Supplementary Table S2, number 99) identified as *Tetranychus evansi* by morphological analysis was identified as a member of order Sarcoptiformes by DNA barcode sequencing. The morphological identification of the adult specimens that were numbered 4 and 82 differed with the identification by DNA barcode sequencing (Supplementary Table S2). These discrepancies are likely due to the analysis of different individuals by two different methods, as many of the vials used in this project contained multiple specimens. 

In most cases, *COI* barcode sequencing using MinION supported the early determination of entomologists and provides a supplementary tool to identify immature life stages, such as eggs and nymphs. However, the *COI* barcode gene is unable to discriminate between some species and alternative barcode genes may offer greater refinement [32]. The possibility of species level identification of small invertebrates using multiple barcodes allows them to be further investigated; however, this may depend on the availability of species-specific sequences available in the public databases. Multiple barcodes can be used in parallel or as a single barcode for family or genus level identification, and another one or more barcodes can be used for species level identification [33]. Most of the well-characterised invertebrates were identified to lower taxonomic levels, as reliable reference sequences are present in available databases. However, many small invertebrates (<0.5 mm in length) belong to taxonomic groups that have not been well-studied and have a limited availability of species-specific reference sequences. The results suggest that generating reference sequences from morphologically characterised specimens is important for implementing this method across a range of taxa.

### 3.2. MinION-Based Barcode Sequencing for Surveillance

**Figure 2:**
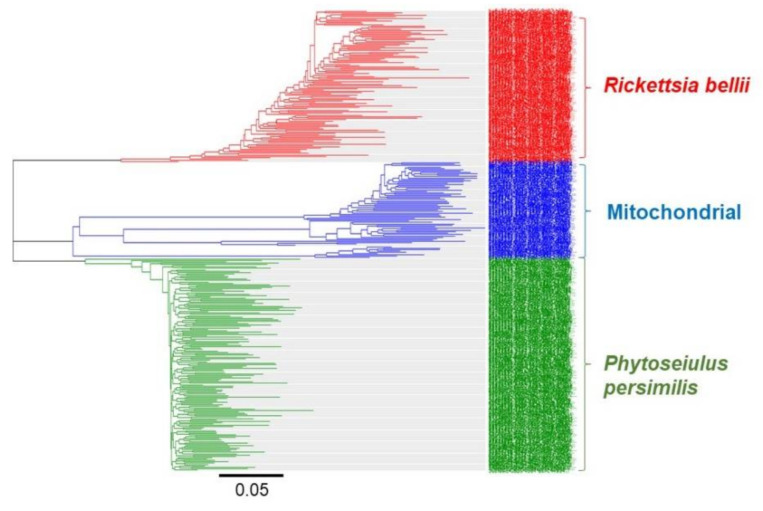
Clustering MinION reads to identify the amplified products from targeted DNA and untargeted contaminated DNA. Different clusters show the DNA barcode (*COI*) amplification from intracellular bacteria *Rickettsia bellii* (red), contaminated human mitochondrial DNA (blue) and mite (*Phytoseiulus persimilis*) (green).

MinION is the smallest and most user-friendly sequencing platform currently available and can run via a USB connected to a standard computer. Recent studies have shown that the portability of MinION makes DNA barcode sequencing possible even in operational settings [10,13]. As an emerging sequencing platform, the price of sequencing using MinION is still higher than previous sequencing platforms. However, the cost per sample can be significantly reduced by multiplexing samples and using more cost-efficient Flongle flow cells. In this study, multiplexing 12–24 specimens in a single Flongle flow cell generated over 5000 reads per specimen. During the early development of MinION sequencing, the higher error rate compared to the other sequencing platforms was identified as a major concern of using MinION in DNA barcoding. This problem was overcome via the development of appropriate analysis workflows, such as ONTrack and SAIGA [13,34]. The clustering of MinION reads into groups allowed amplified products from the specimen to be identified from non-targeted template DNA. For example, Figure 2 shows the amplification of the *COI* barcode not only from the specimens but also from intracellular bacteria *Rickettsia bellii* and contaminant human mitochondrial DNA. It is important to exclude the amplified products from untargeted DNA in order to generate high accuracy consensus sequences from the specimen. On the other hand, clustering *COI* barcode reads can be a good approach to identify the vectors of invertebrate species carrying pathogenic bacteria that can cause diseases to humans and animals. The results showed that after sorting the reads by length and clustering into groups using a Geneious Prime bioinformatics software platform, only 100–150 individual reads are required to generate high-accuracy consensus sequences. 

**Figure 3:**
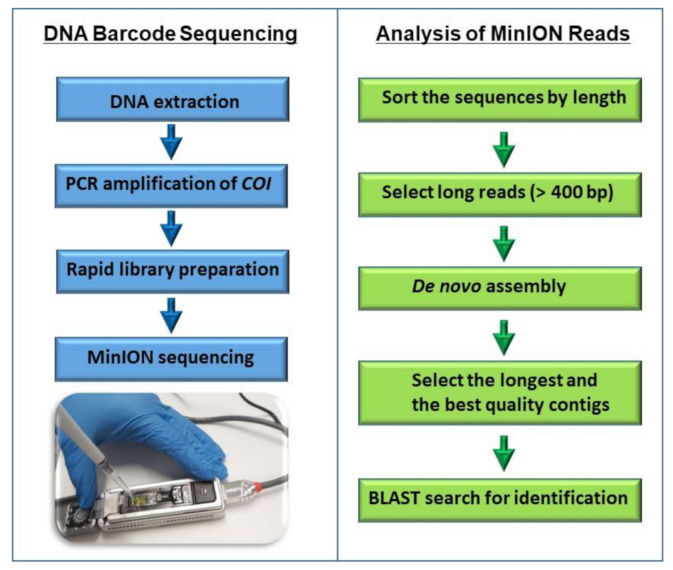
A rapid DNA barcode sequencing workflow for identification of invertebrates.

In this study, the suitability of the ONT rapid sequencing principle for sequencing the PCR-amplified *COI* barcode was also assessed. The rapid sequencing method includes a transposase for fragmenting the amplified *COI* barcode and the rapid addition of sequencing adapters. This method produced reads in various lengths due to the fragmentation by transposase, but after de novo assembly of the reads using Geneious Prime bioinformatics software platform, the longest and best quality contigs showed a high percentage (>97%) identity to the matching reference record in the NCBI database. This method allowed for the rapid sequencing (10 min sample preparation) of the PCR-amplified *COI* barcode without the requirement of expensive and time consuming AMPure bead purification steps. The steps of the rapid DNA barcode sequencing workflow are shown in Figure 3. In order to validate the rapid barcode sequencing approach, ten specimens (Table 3) previously identified to species level using the ONT ligation sequencing kit (SQK-LSK109) were further analysed using the ONT rapid sequencing kit (SQK-RAD004) and their identity was confirmed with an over 97% sequence identity to the closest matching reference record.

Our results indicate that a MinION-based DNA barcode sequencing workflow can be further improved for use in operational settings. The generation of custom databases allows the user to add new barcode sequences for the consecutive identification of the same species, and to overcome the disadvantages of using public databases. This would eliminate the likelihood of hits to incorrect entries of species names and the lack of data movement from private to public databases [35]. The barcoding capacity can be further improved by using the ONT Rapid Barcoding Sequencing (SQK-RBK004) kit and also via tagged amplicon sequencing to introduce 13–20 bp tags in PCR-amplified products [11] and demultiplexing using an appropriate bioinformatics pipeline [12]. Sequencing amplicons from mixed samples and clustering sequences in order to generate consensus sequences from each cluster are also possible in reducing time and cost for library preparations. Recently, ONT have introduced numerous workflows, such as Ultra-long DNA Sequencing (SQK-ULK001) and Cas9 Targeted sequencing; however, the suitability of such protocols for biosecurity needs to be further investigated.

### 3.3. Comparison of MinION Rapid Barcode Sequencing to Existing Sangar Sequencing and Morphological Identification

No consistent differences were observed between Sanger and MinION consensus reads of the same sample. Both sequencing technologies showed consistent results. For example, the identification of eight specimens using both Sanger and MinION sequencing of the *COI* barcode is shown in Table 4. Seven out of eight specimens were identified with over 98% sequence identity from both methods. Both MinION and Sanger sequencing results showed low sequence identity (77–79%) for one specimen (Table 4, number 7) and highlighted the gaps in public databases as a limiting factor for sequence-based identification by either sequencing technology. 

In this study, the cost benefit vs. time analysis comparing MinION rapid barcode sequencing to existing Sangar sequencing and morphological identification was conducted. The time taken to obtain results and the cost between different diagnostic methods can be variable between different laboratories. However, Sanger sequencing might be the most time-consuming method, as it requires postage of the amplified products to a third-party service provider and a wait period in order to retrieve the results. According to the estimate provided by DAWE entomologists, the time taken to retrieve Sanger sequencing results is around five days, and the average time to identify a single specimen using morphological/microscopic tools is around 30 min, noting that time inputs vary between specimens (Supplementary Table S3). Both microscopic and MinION sequencing methods take the same amount of time (4 h) to identify eight specimens. However, the morphological identification of 12 specimens using a microscope can take around 6 h. This is more time consuming compared to using the MinION rapid barcode sequencing method to sequence the same number of specimens (12 specimens takes 4.6 h). In terms of cost, MinION sequencing-based identification of eight specimens is approximately four times cheaper than microscopic identification. Overall, the results of the cost benefit vs. time analysis showed that MinION rapid barcode sequencing is the most cost-effective option for the analysis of four or more specimens.

## 4. Conclusions

This study shows a side-by-side comparison of the DNA barcode sequencing-based identification and the morphological identification of a variety of large and small invertebrate specimens intercepted at the Australian border. Most egg and immature stages that are difficult to identify by morphological analysis were identified to lower taxonomic levels, such as genus and species, by DNA barcode sequencing using MinION. This study also showed that sequencing *COI* barcodes using the ONT rapid sequencing principle is a cost-effective and field-adaptable sequencing approach. Overall, the results of this study suggest the importance of incorporating DNA barcode sequencing using MinION with morphological identification into border diagnostic programs. Initially, MinION-based DNA barcode sequencing may require the establishment of custom databases as DNA barcode reference libraries. This is an ongoing process, but utilising MinION sequencing technology will offer a complementary approach to morphology-based identification, enabling better informed biosecurity decision making and providing a significantly faster and cost-effective alternative to the existing Sanger sequencing molecular identification process.

## Figures and Tables

**Table 1:** Primers used to amplify DNA fragments of the COI gene.

Primer Name	Primer Sequence
LCO1490 (Forward)	5′-GGTCAACAAATCATAAAGATATTGG-3′
HC02198 (Reverse)	5′-TAAACTTCAGGGTGACCAAAAAATCA-3′

**Table 2:** A side-by-side comparison of DNA barcode sequence-based identification and the morphological identification.

Number	Developmental Stage	Initial Determination by Microscopic Methods	Determination by MinION Barcode Sequencing	Comments
1	Adult	*Trogoderma*	*Trogoderma variabile*	Improved BDM*
			98.48% Id*	
2	Adult	*Trogoderma*	*Trogoderma anthrenoides*	Improved BDM*
			97.89% Id*	
3	Adult	*Beetle*	*Trogoderma granarium*	Improved BDM*
			97.92% Id*	
4	Adult	*Trogoderma granarium*	*Trogoderma granarium*	Supported BDM*
			98.37% Id*	
5	Adult	*Franklinella occidentalis*	*Franklinella occidentalis*	Supported BDM*
			98.05% Id*	
6	Larva	*Thripidae*	*Franklinella occidentalis*	Improved BDM*
			98.39% Id*	
7	Adult	*Franklinella shultzei*	*Franklinella shultzei*	Supported BDM*
			98.05% Id*	
8	Larva	*Thripidae*	*Franklinella occidentalis*	Improved BDM*
			99.55% Id*	
9	Larva	*Thripidae*	*Franklinella occidentalis*	Improved BDM*
			98.35% Id*	
10	Adult	*Franklinella occidentalis*	*Franklinella occidentalis*	Supported BDM*
			99.55% Id*	
11	Adult	*Franklinella occidentalis*	*Franklinella occidentalis*	Supported BDM*
			99.10% Id*	
12	Adult	*Franklinella occidentalis*	*Franklinella occidentalis*	Supported BDM*
			99.70% Id*	
13	Adult	*Franklinella shultzei*	*Franklinella shultzei*	Supported BDM*
			98.63% Id*	
14	Adult	*Franklinella shultzei*	*Franklinella shultzei*	Supported BDM*
			98.25% Id*	
15	Adult	*Franklinella occidentalis*	*Franklinella occidentalis*	Supported BDM*
			98.86% Id*	
16	Adult	*Franklinella occidentalis*	*Franklinella occidentalis*	Supported BDM*
			97.68% Id*	

BDM*—Biosecurity decision making (different colours highlight the different BDM outcomes); Id*—Sequence identity percentage.

**Table 3:** A side-by-side comparison of DNA barcode sequence-based identification and the morphological identification of ten invertebrate specimens intercepted at the border.

Number	Developmental Stage	Initial Determination by Microscopic Methods	Determination by MinION Sequencing of Barcode	Comments
1	Adult	*Tyrophagus curvipenis*	*Tyrophagus curvipenis*	Supported BDM*
			97.29% Id*	
2	Adult	*Tetranychus urticae*	*Tetranychus urticae*	Supported BDM*
			99.25% Id*	
3	Adult	*Tetranychus ludeni*	*Tetranychus ludeni*	Supported BDM*
			98.96% Id*	
4	Adult	*Tetranychus evansi*	*Aculops lycopersici*	Improved BDM*
			98.67% Id*	
5	Adult	*Acari (Sub-class)*	*Phytoseiulus persimilis*	Improved BDM*
			97.47% Id*	
6	Egg	*Arachnida (Class)*	*Phytoseiulus persimilis*	Improved BDM*
			97.40% Id*	
7	Egg	*Not detected*	*Haplothrips sp*	Improved BDM*
			98.92% Id*	
8	Egg	*Insecta (Class)*	*Phloeonomus punctipennis*	Improved BDM*
			97.19% Id*	
9	Egg	*Not detected*	*Signiphora flavella*	Improved BDM*
			98.93% Id*	
10	Egg	*Blattodea (Order)*	*Choristima sp*	Improved BDM*
			99.24% Id*	

BDM*—Biosecurity decision making (different colours highlight the different BDM outcomes); Id*—Sequence identity percentage.

**Table 4:** A side-by-side comparison of identification results from Sanger and MinION sequencing technologies.

Number	Determination by Sanger Sequencing of Barcode	Determination by MinION Sequencing of Barcode	Database
1	*Bactrocera xanthodes* 99.88% Id*	*Bactrocera xanthodes* 99.24% Id*	NCBI

2	*Aleuroctarthrus destructor* 99.34% Id*	*Aleuroctarthrus destructor* 99.38% Id*	BOLD

3	*Lepidoptera* 100% Id*	*Lepidoptera* 100% Id*	BOLD

4	*Spodoptera litura* 100% Id*	*Spodoptera litura* 100% Id*	BOLD

5	*Liriomyza trifolii* 100% Id*	*Liriomyza trifolii* 99.64% Id*	BOLD

6	*Helicoverpa armigera* 99.85% Id*	*Helicoverpa armigera* 98.93% Id*	NCBI

7	*Aleyrodidae* 78.64% Id*	*Aleyrodidae* 77.4% Id*	BOLD

8	*Helicoverpa armigera* 100% Id*	*Helicoverpa armigera* 100% Id*	BOLD


Id*—Sequence identity percentage.

## Supplementary Materials

The following are available online at https://www.mdpi.com/article/10.3390/genes12081138/s1, Table S1: A side-by-side comparison of DNA barcode sequencing-based identification of khapra beetle and thrips specimens intercepted at the border, Table S2: A side-by-side comparison of DNA barcode sequencing-based identification of invertebrate specimens intercepted at the border, Table S3: Cost-benefit versus time analysis comparing MinION sequencing to existing diagnostics.
