# Supplementary material for: A Rapid and Cost-Effective Identification of Invertebrate Pests at the Borders Using MinION Sequencing of DNA Barcodes
Source: Genes (Basel). 2021 Jul 27;12(8):1138. doi: 10.3390/genes12081138 (PMC8392835; doi:10.3390/genes12081138)
Supplement: Supplementary file 1 [file genes-12-01138-s001.zip › Supplementary Table S1.pdf]

Supplementary Table S1. A side-by-side comparison of DNA barcode sequencing-based identification and the morphological identification of khapra beetle and thrips specimens intercepted at the border.

Number	Specimen ID	Developmental stage	Initial determination by microscopic methods	Barcode sequencing; The best blast hit in NCBI database	Sequence Identity (%)	NCBI Accession number	MinION sequencing ID compared to morphological			
							**Support morphological ID to the taxonomic level**	**Family/genus level identification (≥90%)**	**Species level identification (≥97%)**	**Comments**
1	274627	Adult	Trogoderma	*Trogoderma variabile*	98.48%	MG458973.1	Genus	Yes	Yes	Improved BDM
2	SA16011589	Adult	Trogoderma	*Trogoderma anthrenoides*	97.89%	KP331485.1	Genus	Yes	Yes	Improved BDM
3	175479	Adult	Beetle	*Trogoderma granarium*	97.92%	KP331467.1	N/A	Yes	Yes	Improved BDM
4	313163	Adult	*Trogoderma granarium*	*Trogoderma granarium*	98.37%	KP331467.1	Species	Yes	Yes	Supported BDM
5	420106	Adult	*Franklinella occidentalis*	*Franklinella occidentalis*	98.05%	MF993426.1	Species	Yes	Yes	Supported BDM
6	398949	Larva	Thripidae	*Franklinella occidentalis*	98.39%	HQ697596.1	Family	Yes	Yes	Improved BDM
7	423419	Adult	*Franklinella shultzei*	*Franklinella shultzei*	98.05%	KF778777.1	Species	Yes	Yes	Supported BDM
8	320598	Larva	Thripidae	*Frankliniella occidentalis*	99.55%	HQ697596.1	Family	Yes	Yes	Improved BDM
9	320599	Larva	Pupa	*Frankliniella occidentalis*	98.35%	MF993426.1	Family	Yes	Yes	Improved BDM
10	420107	Adult	*Frankliniella occidentalis*	*Frankliniella occidentalis*	99.55%	HQ697596.1	Species	Yes	Yes	Supported BDM
11	423417	Adult	*Frankliniella occidentalis*	*Frankliniella occidentalis*	99.10%	HQ697596.1	Species	Yes	Yes	Supported BDM
12	320600	Adult	*Frankliniella occidentalis*	*Frankliniella occidentalis*	99.70%	HQ697596.1	Species	Yes	Yes	Supported BDM
13	111111	Adult	*Frankliniella schultzei*	*Frankliniella schultzei*	98.63%	KP871483.1	Species	Yes	Yes	Supported BDM

14	111111	Adult	*Frankliniella schultzei*	*Frankliniella schultzei*	98.25%	KC513059.1	Species	Yes	Yes	Supported BDM
15	222222	Adult	*Frankliniella occidentalis*	*Frankliniella occidentalis*	98.86%	KC513060.1	Species	Yes	Yes	Supported BDM
16	423417	Adult	*Frankliniella occidentalis*	*Frankliniella occidentalis*	97.68%	GQ343257.1	Species	Yes	Yes	Supported BDM

BDM – Biosecurity Decision Making.
