# Supplementary material for: A Rapid and Cost-Effective Identification of Invertebrate Pests at the Borders Using MinION Sequencing of DNA Barcodes
Source: Genes (Basel). 2021 Jul 27;12(8):1138. doi: 10.3390/genes12081138 (PMC8392835; doi:10.3390/genes12081138)
Supplement: Supplementary file 1 [file genes-12-01138-s001.zip › Supplementary Table S2.pdf]

Supplementary Table S2. A side-by-side comparison of DNA barcode sequencing-based identification and the morphological identification of invertebrate specimens intercepted at the border.

Number	Specimen ID	Developmental stage	Initial determination by microscopic methods	Barcode sequencing; The best blast hit in NCBI database	Sequence Identity (%)	NCBI Accession number	MinION sequencing ID compared to morphological			
							Support morphological ID to the taxonomic level	Family/genus level identification (≥90%)	Species level identification (≥97%)	Comments
1	411850	Adult	*Tyrophagus curvipenis*	*Tyrophagus curvipenis*	97.29%	KY986272.1	Species	Yes	Yes	Supported BDM
2	1XXXX	Adult	*Tetranychus urticae*	*Tetranychus urticae*	99.25%	KJ729022.1	Species	Yes	Yes	Supported BDM
3	4XXXX	Adult	*Tetranychus ludeni*	*Tetranychus ludeni*	98.96%	KJ729018.1	Species	Yes	Yes	Supported BDM
4	2XXXX	Adult	*Tetranychus evansi*	*Aculops lycopersici*	98.67%	JX298841.1	Order: Prostigmata	Yes	Yes	Improved BDM
5	359697	Adult	Acari (Sub-class)	*Phytoseiulus persimilis*	97.47%	KY271881.1	Sub-class	Yes	Yes	Improved BDM
6	396771	Egg	Class: Arachnida	*Phytoseiulus persimilis*	97.40%	KY271881.1	Class	Yes	Yes	Improved BDM
7	393706_2	Egg	Not detected	*Haplothrips sp*	98.92%	MG359779.1	N/A	Yes	No	Improved BDM
8	359688	Egg	*Arthropoda, possibly Insecta*	*Phloeonomus punctipennis*	97.19%	JX626112.1	Class	Yes	Yes	Improved BDM
9	396687	Egg	No ID	*Signiphora flavella*	98.93%	MH456558.1	N/A	Yes	Yes	Improved BDM
10	411231	Egg	*Order: Blattodea*	*Choristima sp*	99.24%	HQ583912.1	Order	Yes	No	Improved BDM
11	397935	Egg	Sub-class: Acari	*Coscinoptycha improbana*	99.24%	HQ921885.1	Phylum: Arthropoda	Yes	Yes	Improved BDM
12	398164	Egg	Sub-class: Acari	Order: Psocoptera sp	99.39%	KM528369.1	Phylum: Arthropoda	No	No	Improved BDM
13	393594	Egg	Class: Arachnida	Order: Psocoptera sp	98.64%	KM528369.1	Phylum: Arthropoda	No	No	Improved BDM
14	401751-3	Egg	No ID	Order: Psocoptera sp	98.33%	KM528369.1	N/A	No	No	Improved BDM
15	396687-1	Adult	Family: Tydeidae	Family: Tydeidae	95.25%	KP979115.1	Family	Yes	No	Supported BDM
16	396687-1	Egg	Family: Tydeidae	Family: Tydeidae	95%	KP979115.1	Family	Yes	No	Supported BDM

17	396687	Nymph	Family: Tydeidae	Family: Tydeidae	95.05%	KP979115.1	Family	Yes	No	Supported BDM
18	396687	Nymph	Family: Tydeidae	Family: Tydeidae	95.28%	KP979115.1	Family	Yes	No	Supported BDM
19	394401	Egg	Family: Tydeidae	Family: Tydeidae	94.80%	KP979115.1	Family	Yes	No	Supported BDM
20	396677	Nymph	Family: Tydeidae	Family: Tydeidae	92.09%	KP979115.1	Family	Yes	No	Supported BDM
21	396677	Nymph	Family: Tydeidae	Family: Tydeidae	93.72%	KP979115.1	Family	Yes	No	Supported BDM
22	328988	Egg	Class: Insecta	*Genus: Neonetus*	90.00%	MT011505.1	Class	Yes	No	Improved BDM
23	336769-1	Egg	Family: Miridae	*Genus: Diomocoris*	93.55%	MH359335.1	Family	Yes	No	Improved BDM
24	358369-2	Egg	Family: Miridae	*Genus: Diomocoris*	93.28%	MH359335.1	Family	Yes	No	Improved BDM
25	358369-3	Egg	Family: Miridae	*Genus: Diomocoris*	93.76%	MH359335.1	Family	Yes	No	Improved BDM
26	396679	Egg	Family: Miridae	*Genus: Diomocoris*	93.91%	MH359335.1	Family	Yes	No	Improved BDM
27	400756-1	Egg	Family: Miridae	*Genus: Diomocoris*	94.22%	MH359335.1	Family	Yes	No	Improved BDM
28	400756-2	Egg	Family: Miridae	*Genus: Diomocoris*	93.75%	MH359335.1	Family	Yes	No	Improved BDM
29	341943	Egg	No ID	*Genus: Diomocoris*	94.38%	MH359335.1	N/A	Yes	No	Improved BDM
30	400756	Egg	No ID	*Genus: Diomocoris*	94.08%	MH359335.1	N/A	Yes	No	Improved BDM
31	397926	Egg	No ID	Genus: *Poecilolycia*	90.43%	HM374185.1	N/A	Yes	No	Improved BDM
32	399630	Egg	No ID	Order: Psocoptera	93.62%	KM528369.1	N/A	No	No	Improved BDM
33	398164	Egg	No ID	Genus: *Xenylla*	94.68%	KT808349.1	N/A	Yes	No	Improved BDM
34	375975	Egg	Class: Insecta	*Genus: Brachycaudus*	94.95%	KR037740.1	Class	Yes	No	Improved BDM
35	376244	Egg	Class: Insecta	*Genus: Ricania*	85.71%	MF621872.1	Class	No	No	CMRR < 90% sequence identity
36	338510	Egg	No ID	*Genus: Neonetus*	89.17%	MT011505.1	N/A	No	No	CMRR < 90% sequence identity
37	412000	Egg	Class: Insecta	*Genus: Thrips*	79.11%	KF144125.1	Class	No	No	CMRR < 90% sequence identity
38	399939	Egg	Family: Miridae	Genus: Orius	84.71%	MG874953.1	Order: Hemiptera	No	No	CMRR < 90% sequence identity
39	394787	Egg	Class: Arachnida	*Genus: Phytoseiulus*	88.86%	KY271881.1	Class	No	No	CMRR < 90% sequence identity
40	359694	Adult	Order: Mesostigmata	Family: Melicharidae	81.78%	MF915504.1	Order	No	No	CMRR < 90% sequence identity

41	394406	Adult	*Genus: Tarsonemus*	Family: Tarsonemidae	84.05%	MG314339.1	Family	No	No	CMRR < 90% sequence identity
42	396887	Egg	Sub-class: Acari	*Genus: Triophtydeus*	82.83%	MG310675.1	Sub-class	No	No	CMRR < 90% sequence identity
43	401751	Egg-1	Sub-class: Acari	*Genus: Amblyseius*	81.34%	JX080327.1	Sub-class	No	No	CMRR < 90% sequence identity
44	401650	Adult	*Genus: Brachytydeus*	Family: Eupodidae	79.76%	JX834220.1	Family	No	No	CMRR < 90% sequence identity
45	383726	Egg	Class: Arachnida	*Genus: Phytoseiulus*	87.50%	KY271881.1	Class	No	No	CMRR < 90% sequence identity
46	401751	Egg-2	Sub-class: Acari	Genus: Amblyseius	82.28%	JX080348.1	Sub-class	No	No	CMRR < 90% sequence identity
47	396899-1	Adult	Family: Tydeidae	Family: Tydeidae	80.31%	KM837940.1	Family	No	No	CMRR < 90% sequence identity
48	396899-2	Adult	Family: Tydeidae	Family: Tydeidae	80.03%	KM837940.1	Family	No	No	CMRR < 90% sequence identity
49	396884	Adult	Sub-order: Oribatida	Family: Oribatulidae	82.25%	MG410173.1	Order: Sarcoptiformes	No	No	CMRR < 90% sequence identity
50	412151-1	Adult	Sub-order: Oribatida	Family: Oribatulidae	82.35%	MG410173.1	Order: Sarcoptiformes	No	No	CMRR < 90% sequence identity
51	412151-2	Egg	Sub-order: Oribatida	Order: Sarcoptiformes	81.27%	KM623317.1	Order: Sarcoptiformes	No	No	CMRR < 90% sequence identity
52	396680	Adult	Order: Sarcoptiformes	Family: Oribatulidae	81.66%	MG410173.1	Order	No	No	CMRR < 90% sequence identity
53	412159-1	Adult	*Genus: Reductobates*	Family: Oribatulidae	82.36%	KJ087519.1	Family	No	No	CMRR < 90% sequence identity
54	412159-2	Adult	Family: Oribatulidae	Family: Oribatulidae	81.75%	KJ087519.1	Family	No	No	CMRR < 90% sequence identity
55	412158	Adult	No ID	*Family: Oribatellidae*	80.55%	MN352316.1	N/A	No	No	CMRR < 90% sequence identity
56	410633-1	Adult	*Genus: Reductobates*	Order: Sarcoptiformes	81.61%	KM623317.1	Order	No	No	CMRR < 90% sequence identity
57	410633-2	Adult	No ID	*Genus: Oppiidae*	81.36%	MG947348.1	N/A	No	No	CMRR < 90% sequence identity

58	393706_1	Nymph	Order: Sarcoptifomes	Order: Sarcoptiformes	82.41%	KM623317.1	Order	No	No	CMRR < 90% sequence identity
59	394408	Adult	Sub-order: Oribatida	Family: Eremaeidae	81.72%	MN347670.1	Order: Sarcoptiformes	No	No	CMRR < 90% sequence identity
60	396884	Adult	Sub-order: Oribatida	Family: Oribatulidae	81.94%	MG410173.1	Order: Sarcoptiformes	No	No	CMRR < 90% sequence identity
61	394854	Adult	Order: Sarcoptifomes	Family: Eremaeidae	81.97%	MN347670.1	Order	No	No	CMRR < 90% sequence identity
62	396774	Nymph	Sub-order: Oribatida	Order: Sarcoptifomes	80.28%	KM623317.1	Order	No	No	CMRR < 90% sequence identity
63	396677	Egg	Class: Arachnida	*Genus: Amblyseius*	81.00%	MN359647.1	Class	No	No	CMRR < 90% sequence identity
64	394401	Nymph	*Genus: Neocalvolia*	*Genus: Nycteridocaulus*	85.82%	KU203210.1	Sub Order: Astigmata	No	No	CMRR < 90% sequence identity
65	396686	Nymph	Sub-order: Oribatida	Order: Sarcoptiformes	80.86%	KM623317.1	Order	No	No	CMRR < 90% sequence identity
66	411835	Adult	Sub-order: Oribatida	Family: Oribatulidae	82.20%	MG410173.1	Order: Sarcoptiformes	No	No	CMRR < 90% sequence identity
67	396679	Adult	Order: Sarcoptiformes	*Genus: Nycteridocaulus*	85.59%	KU203210.1	Order	No	No	CMRR < 90% sequence identity
68	342022	Adult	Sub-order: Oribatida	Super-family: Eremaeidae	81.94%	MN347670.1	Sub-order	No	No	CMRR < 90% sequence identity
69	396678	Adult	Order: Sarcoptiformes	Family: Oribatulidae	81.86%	MG410173.1	Order	No	No	CMRR < 90% sequence identity
70	330873_1	Adult	Sub-order: Oribatida	*Genus: Peloribates*	83.31%	MN348941.1	Sub-order	No	No	CMRR < 90% sequence identity
71	394792	Adult	Sub- order: Oribatida	Family: Oribatulidae	82.02%	MG410173.1	Sub-order	No	No	CMRR < 90% sequence identity
72	330873_2	Adult	Sub-order: Oribatida	*Genus: Toxerodectes*	82.00%	KU203236.1	Order: Sarcoptiformes	No	No	CMRR < 90% sequence identity
73	396773	Nymph	Sub-order: Oribatida	Family: Eremaeidae	80.67%	MN347670.1	Sub-order	No	No	CMRR < 90% sequence identity
74	394443	Nymph	Order: Sarcoptiformes	Family: Oribatulidae	80.41%	MG410173.1	Order	No	No	CMRR < 90% sequence identity

75	393594	Adult	Sub-order: Oribatida	*Genus: Neoribates*	87.31%	MK015000.1	Sub-order	No	No	CMRR < 90% sequence identity
76	323546	Adult	Order: Sarcoptiformes	Family: Eremaeidae	81.62%	MN347670.1	Order	No	No	CMRR < 90% sequence identity
77	399949	Adult	*Genus: Reductobates*	*Order: Sarcoptiformes*	82.36%	KM623317.1	Order	No	No	CMRR < 90% sequence identity
78	399629	Adult	Sub-order: Oribatid	*Genus: Zetomimus*	85.56%	MN353928.1	Sub-order	No	No	CMRR < 90% sequence identity
79	342018-1	Adult	Sub-order: Oribatida	*Genus: Neoribates*	80.64%	MN360340.1	Sub-order	No	No	CMRR < 90% sequence identity
80	342018-2	Adult	Sub-order: Oribatida	Family: Oribatulidae	82.5%	KJ087519.1	Sub-order	No	No	CMRR < 90% sequence identity
81	393594	Adult	Sub-order: Oribatida	Family: Oribatulidae	82.79%	MG410173.1	Sub-order	No	No	CMRR < 90% sequence identity
82	399630	Adult	Sub-order Oribatid	*Genus: Eueremaeus*	82.89%	JX838540.1	Sub-order	No	No	CMRR < 90% sequence identity
83	399618	Adult	No ID	*Genus: Xolalgoides*	85.32%	KU203106.1	N/A	No	No	CMRR < 90% sequence identity
84	400552	Adult	No ID	Family: Oribatulidae	81.78%	MG410173.1	N/A	No	No	CMRR < 90% sequence identity
85	399374	Egg	Class: Arachnida	Family: Oribatulidae	81.76%	KJ087519.1	Class	No	No	CMRR < 90% sequence identity
86	396890	Adult	Sub-order: Oribatida	Family: Oribatulidae	82.62%	KJ087519.1	Sub-order	No	No	CMRR < 90% sequence identity
87	396772	Adult	Sub-order: Oribatida	Family: Oribatulidae	82.30%	KJ087519.1	Sub-order	No	No	CMRR < 90% sequence identity
88	383727	Adult	Sub-order: Oribatida	Super family: Eremaeoidea	80.85%	MG417012.1	Sub-order	No	No	CMRR < 90% sequence identity
89	396772	Adult	Sub-order: Oribatida	Family: Oribatulidae	82.07%	MG410173.1	Sub-order	No	No	CMRR < 90% sequence identity
90	358910	Adult	Family: Oribatulidae	Family: Oribatulidae	82.62%	KJ087519.1	Family: Oribatulidae	No	No	CMRR < 90% sequence identity
91	393594	Adult	Sub-order: Oribatida	Order: Sarcoptiformes	82.48%	KM623317.1	Order	No	No	CMRR < 90% sequence identity

92	383727-1	Adult	Sub-order: Oribatida	Family: Oribatulidae	82.77%	KJ087608.1	Sub-order	No	No	CMRR < 90% sequence identity
93	383727-2	Adult	Sub-order: Oribatida	Family: Oribatulidae	83.59%	MG410173.1	Sub-order	No	No	CMRR < 90% sequence identity
94	396772	Adult	Sub-order: Oribatida	Order: Sarcoptiformes	81.71%	KM834060.1	Order	No	No	CMRR < 90% sequence identity
95	342007	Adult	Sub-order: Oribatida	Order: Sarcoptiformes	81.92%	KM623317.1	Order	No	No	CMRR < 90% sequence identity
96	396771	Adult	Order: Sarcoptiformes	*Genus: Neoribates*	87.10%	MK015000.1	Order	No	No	CMRR < 90% sequence identity
97	342018	Adult	Sub-order: Oribatida	Family: Oribatulidae	82.46%	KJ087519.1	Sub-order	No	No	CMRR < 90% sequence identity
98	412151	Adult	Sub-order: Oribatida	Family: Oribatulidae	82.77%	KJ087519.1	Sub-order	No	No	CMRR < 90% sequence identity
99	S386142	Egg	Species: *Tetranychus evansi*	Family: Oribatulidae	82.24%	KJ087519.1	Superorder: Acariformes	No	No	CMRR < 90% sequence identity
100	399630	Adult	Sub-order: Oribatid	Family: Ascidae	82.68%	MG412078.1	Sub-class: Acari	No	No	CMRR < 90% sequence identity

BDM – Biosecurity Decision Making.

CMRR – Closest Matching Reference Record
