# Supplementary material for: A Rapid and Cost-Effective Identification of Invertebrate Pests at the Borders Using MinION Sequencing of DNA Barcodes
Source: Genes (Basel). 2021 Jul 27;12(8):1138. doi: 10.3390/genes12081138 (PMC8392835; doi:10.3390/genes12081138)
Supplement: Supplementary file 1 [file genes-12-01138-s001.zip › Supplementary Table S3.pdf]

Supplementary Table S3. Cost-benefit versus time analysis comparing MinION sequencing to existing diagnostics.

	**1 X Specimen**		**4 X Specimen**		**6 X Specimen**		**8 X Specimen**		**12 X Specimen**	
	Time (hrs)	Cost (AUD)	Time (hrs)	Cost (AUD)	Time (hrs)	Cost (AUD)	Time (hrs)	Cost (AUD)	Time (hrs)	Cost (AUD)
**Microscopic***	0.5	$ 100.00	2	$ 400.00	3	$ 600.00	4	$ 800.00	6	$ 1200.00
**Sanger**	120	$ 74.86	120	$ 170.83	120	$ 234.81	120	$ 298.79	120	$ 426.75
**MinION**	3.1	$ 210.29	3.5	$ 151.25	3.8	$ 178.55	4	$ 212.53	4.6	$ 287.00

*Average time estimate provided by DAWE entomologist noting time inputs varies between specimens.
